# A Novel PTS Technique with Side Information Blind Detector Based on Minimized Error Accumulation for PAPR Reduction in Coded Underwater Acoustic OFDM Systems

**DOI:** 10.3390/s25185763

**Published:** 2025-09-16

**Authors:** Siyu Xing, Bo Wei, Yanting Yu, Yiqi Bai, Jiawei Yin

**Affiliations:** 1 State Key Laboratory of Physical Oceanography, Institute of Oceanographic Instrumentation, Qilu University of Technology (Shandong Academy of Sciences), Qingdao 266100, China; xingsiyu@qlu.edu.cn (S.X.); yuyanting@qlu.edu.cn (Y.Y.); baiyiqi@qlu.edu.cn (Y.B.); yinjiawei@qlu.edu.cn (J.Y.); 2 Laoshan Laboratory, Qingdao 266237, China

**Keywords:** underwater acoustic communication (UAC), orthogonal frequency division multiplexing (OFDM), partial transmit sequence (PTS) technique, peak-to-average power ratio (PAPR) reduction, side information

## Abstract

In this paper, a partial transmit sequence (PTS) technique with a side information (SI) blind detector based on minimized error accumulation for PAPR reduction in the coded underwater acoustic (UWA) orthogonal frequency division multiplexing (OFDM) communication system is proposed. Due to the complexity of UWA channels, channel coding is inevitably employed to ensure accurate information transmission. However, the error correction capability of channel coding usually has a certain margin; the decoder will fail to provide the expected coding gain for the system when the number of error bits exceeds the error correction capability. Therefore, by utilizing the accumulation impact of bit errors inherent in channel coding, it can be regarded as an indicator vector for the SI blind detector, enabling autonomous identification of the weighted phase factor vector sequence index without prior information. Thus, there is no need to reserve the SI transmission symbols, as required by the conventional PTS (C-PTS) scheme, effectively reducing the probability of large-scale bit error caused by the wrong SI. The experimental results show that the BER performance of the system has seen an improvement of about one order of magnitude compared to C-PTS. Consequently, the proposed technique enhances system bandwidth utilization and communication efficiency, ensuring the real-time performance of the underwater acoustic communication (UAC) OFDM system.

## 1. Introduction

Underwater acoustic (UWA) channels are among the most challenging communication media due to their highly complex propagation environment. They exhibit time-, frequency-, and space-selective fading caused by turbulence, multipath effects, and environmental heterogeneity, respectively. Additional impairments include time-varying Doppler shifts, high ambient noise, substantial transmission loss, and narrow bandwidth. These factors collectively lead to non-stationary and spatio-temporally variant channel behavior, making reliable high-rate communication particularly difficult [1,2,3].

Orthogonal frequency division multiplexing (OFDM) has emerged as a key technique for high-speed UWA communications owing to its robustness against multipath interference, efficient spectral utilization, and adaptability to networking applications [4,5,6]. However, a significant limitation of OFDM is its high peak-to-average power ratio (PAPR), resulting from the coherent superposition of multi-carrier signals. High PAPR causes nonlinear operation in power amplifiers and other components, leading to signal distortion, inter-carrier interference, and increased bit error rates. Mitigating PAPR is thus essential to avoid compromising system efficiency and reliability, especially in power-constrained underwater platforms [7].

PAPR reduction techniques are generally classified into three categories, each with distinct limitations. Signal pre-distortion approaches, such as clipping, are simple and computationally efficient but introduce in-band distortion and out-of-band noise, which can severely degrade bit error rate (BER) under demanding UWA channel conditions. Coding-based schemes enhance reliability through error correction and direct PAPR control. Yet, they suffer from rate loss, limited codebook availability, and high computational overhead, which restricts their use in high-rate, flexible UWA-OFDM systems [8]. Probabilistic methods achieve lossless PAPR reduction but require transmitting side information (SI), increasing redundancy and complexity. Their BER performance is also sensitive to SI recovery accuracy, and additional SI symbols may affect real-time operation in UWA communications.

To overcome the limitations of individual technologies, researchers have tried combining different techniques, such as clipping and filtering, clipping and probabilistic methods (like Clipping-Selected Mapping, Clipping-SLM) [9,10,11], and other integrated algorithms. Banoori proposed a hybrid approach that combines improved repeated frequency-domain filtering and clipping (RFC) with various types of companding techniques to reduce PAPR and improve energy efficiency. Simulation results show that the PAPR reduction exceeds 5 dB (at CCDF = 10^−3^). Moreover, the optimized RFC requires a low number of iterations (≤3 times). The companding operation is computationally simple, making it suitable for resource-constrained underwater devices [12]. However, some of the combined algorithms may worsen their problems simultaneously. The integrated approach usually results in greater overall complexity. For example, nonlinear distortion from clipping can weaken the optimization achieved through probabilistic methods (such as disrupting phase sequence characteristics), leading to limited or no improvements in later processing stages; at the same time, the computational complexity increases [13,14].

Selected mapping (SLM) [15,16,17] and partial transmit sequence (PTS) [18,19,20] are two widely used probabilistic techniques for PAPR reduction in OFDM systems through phase scrambling. They effectively lower PAPR by reducing the probability of high peaks, but require numerous IFFT operations, resulting in computational complexity that increases with the number of scrambling sequences. Improved algorithms aim to strike a better balance between complexity and PAPR reduction. For example, pre-setting a threshold in PTS reduces complexity at the cost of some PAPR performance [21]. A simplified PTS scheme [22] significantly decreases complexity compared to [18], with only slight degradation in PAPR performance; this is referred to as Conventional PTS (C-PTS) in this paper. A pseudo-optimal PTS scheme [23] further reduces the complexity of selecting the best signal in C-PTS. Moreover, it enhances PAPR performance due to random phase vector selection, unlike C-PTS, which uses a fixed set.

However, another disadvantage of conventional SLM and PTS techniques is the need to transmit numerous SI bits to ensure accurate data recovery at the receiver. As a result, the system’s BER performance heavily relies on the accuracy of the SI. Any error in the SI can cause a significant BER for the entire system. To ensure the SI’s accuracy, a specific encoding scheme or multiple transmissions of the SI are often required, but this results in sending a large amount of redundant data. This reduces communication efficiency and wastes bandwidth. Additionally, SI data are usually sent after all other signals, which can create storage issues at the receiver and affect real-time system performance.

There are a considerable number of studies that have been able to provide methods to overcome this shortcoming [24,25,26,27,28,29,30]. The methods can be broadly categorized into two types. One approach involves embedding SI into OFDM symbols that carry transmission data, thereby trading off a small portion of the data rate for real-time performance [24,25,26]. Joo H. S. gave identifiable phase offsets to the elements of each rotating vector, and then embedded the SI-identifying rotating vectors into the alternative signal sequences [24]. In Reference [25], the SI is embedded into data subcarriers before OFDM modulation. Reference [26] utilized the nested code structure of the polar code, embedding the SI when the channel coding is complete. These approaches, which incorporate SI embedding, can mitigate storage constraints on the receiving end; however, the embedded SI not only diminishes spectrum utilization but also poses significant challenges in ensuring transmission accuracy.

The other type considers the channel characteristics, selecting different indicator vectors at the transmitting end, without occupying the actual transmission data rate. It then recovers and reconstructs the SI at the receiving end using a blind SI detector [27,28,29,30]. In reference [28], the channel estimation scheme and the PTS scheme are combined to eliminate the necessity for separate SI transmission. The positions within the data block have been allocated to the SI index, thereby extending the modulation symbols. Additionally, an SI detector identifies the positions of the extended symbols at the receiver, facilitating the completion of SI reconstruction. A consistent drawback was the degradation in channel estimation performance at the subblock boundaries, likely due to inter-subblock interference or reduced pilot density. References [29,30] propose two blind SI detection schemes, considering the characteristics of the UWA channels, which automatically and flawlessly identify the side information. These two algorithms utilize the sparse nature of underwater acoustic channels. At the receiver side, different phase weighting factors are applied for de-weighting, and the channel impulse response is estimated via compressed sensing. The characteristics of this impulse response are then used to recover side information (SI) autonomously. A key advantage of the algorithm is its full incorporation of channel sparsity, enabling seamless integration of channel estimation and SI identification. However, the method is computationally intensive. Each compressed sensing-based channel estimation requires multiple iterations and updates to the atoms. Moreover, as the number of sub-blocks increases, the number of required channel estimations grows rapidly, imposing a substantial computational burden on the receiver.

Based on comprehensive theoretical analysis and practical engineering considerations, the decoding and re-encoding processes of convolutional codes are easier to implement than channel estimation algorithms. For instance, many types of digital signal processors (DSPs) feature built-in convolutional codecs, which offer higher encoding and decoding efficiency compared to other coding schemes and are more widely adopted in real-world applications. Therefore, we propose a PTS technique with an SI blind detector based on minimized error accumulation for PAPR reduction in the coded UWA OFDM communication system. Due to the complexity of UWA channels, channel coding is inevitably used to ensure accurate information transmission. However, the error correction capability of channel coding usually has a certain margin; when the number of error bits exceeds this capability, the decoder will fail to provide the expected coding gain. Therefore, by leveraging the accumulation effect of bit errors inherent in channel coding, it can serve as an indicator vector for the SI blind detector, enabling autonomous identification of the weighted phase factor vector sequence index without prior information. Thus, there is no need to reserve the SI transmission symbols, effectively reducing the probability of large-scale bit errors caused by incorrect SI. Consequently, the proposed technique improves system bandwidth utilization and communication efficiency, ensuring the real-time performance of the UAC OFDM system.

The rest of this paper is organized as follows. Section 2 introduces the error accumulation effect of channel coding. In Section 3, we present a novel SI blind detector based on minimized error accumulation for the PTS scheme. In Section 4, the simulation and field experimental results are presented and thoroughly analyzed, while Section 5 outlines the conclusions.

## 2. The Error Accumulation Effect of Channel Coding

Due to noise in the channel, errors will always occur during information transmission. Currently, channel coding helps reduce these errors. If errors occur in the information bits, the transmitted redundant bits can be used for error correction. Naturally, this error correction ability has a limit. When the number of incorrect information bits exceeds a certain point, the decoder cannot provide the expected coding gain. Among various channel coding methods, convolutional codes have been widely adopted in underwater acoustic communication systems due to their simplicity in encoding and decoding algorithms, as well as their superior coding gain performance. Based on theoretical analysis and practical engineering applications, convolutional codes are easier to implement than low-density parity check (LDPC) codes and polar codes. Many types of digital signal processors have built-in codecs for convolutional codes, and the time required for decoding and re-encoding is significantly shorter than that of other coding methods. Meanwhile, the blind detection indicator vector based on minimizing error accumulation is not suitable for decoding methods that take soft information, such as likelihood ratios, as input, as is the case with polar codes. Instead, methods such as embedding SI can be employed to enable the transmission of SI along with the symbols, thereby ensuring symbol-level real-time performance in UWA OFDM communication systems. Therefore, this paper selects convolutional codes as a representative scheme to investigate a PAPR reduction algorithm that minimizes error accumulation in coded UWA OFDM communication systems without requiring side information transmission. The following section offers a concise overview of error propagation in the decoding process of convolutional codes.

**Figure 1:**
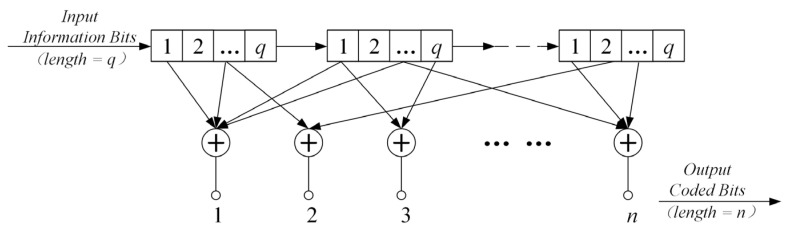
General structure of the convolutional codes.

The general structure of convolutional code encoding is shown in Figure 1, where a small grid represents a shift register that can store 1 bit of information. The information bits flow rightward, driven by the clock.

For a convolutional code that takes q information bits as input and produces n coded bits as output, its shift registers are segmented into groups of length q, amounting to a total of L groups, where L signifies the constraint length. The error-correction capability of convolutional codes heavily relies on their distance characteristics, notably the free distance $d_{free}$, decoding algorithms, and structural formats, including systematic or non-systematic, recursive or non-recursive configurations. The determination of their free distance $d_{free}$ is as follows:

(1) $$d_{free}\leq \left(n-q\right)\cdot \left[1+floor\left(\delta /q\right)\right]+\delta +1,$$

**Figure 2:**
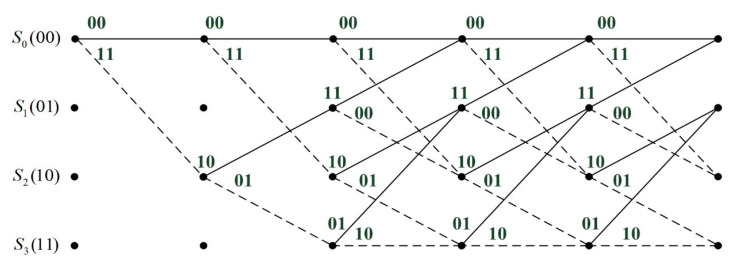
The trellis diagram of the convolutional codes.

The Viterbi decoding process of convolutional codes can also be described using a trellis diagram, which is shown in Figure 2. For convolutional codes, the error-correcting capability t and the free distance $d_{free}$ are related by $t=floor\left(\left(d_{free}-1\right)/2\right)$. If an incorrect decision is made during the decoding process, it means that the decoder’s state has also become erroneous. At this point, the decoder will experience consecutive incorrect decisions. If the quality of subsequent data is good, the decoder still has a chance to return to the correct state. However, suppose the number of consecutive erroneous decisions exceeds the error-correcting capability t. In that case, the entire decoder will fall into a vicious cycle of error accumulation, ultimately leading to an irreversible error disaster.

Therefore, the error accumulation effect present in channel coding can serve as an indicator for the SI blind detector in the PTS algorithm. Assuming that all weighted phase factor vectors used in the PTS algorithm are known a priori to the receiver, the receiver first applies inverse weighting to the received data, then performs de-constellation mapping, decoding, and secondary encoding on the inverse-weighted data. It then compares the data after secondary encoding with the data before decoding. When an incorrect weighted phase factor vector is used for inverse weighting, the persistent errors inherent in the received data will exceed the error correction capability of the convolutional code, leading to an error catastrophe. Conversely, data that have been correctly inverse-weighted will not show any error accumulation. Therefore, this paper proposes a side information blind detection scheme based on minimized error accumulation for the PTS scheme, enabling autonomous identification of the weighted phase factor vector sequence index without prior information. The principle of the PTS technique, with a side information blind detector based on minimizing error accumulation, is discussed in the next section.

## 3. The Principle of the PTS Technique with Side Information Blind Detector Based on Minimized Error Accumulation

At the transmitter, the procedures of subblock partitioning for the data to be transmitted, individually applying M sets of phase factor vector weighting to the partitioned subblocks, and subsequently merging them, align with the C-PTS algorithm. The transmitted data $X_{D}^{m}$ obtained follow the format presented in Equation (2).

(2) $$X_{D}^{m}=\sum_{v=1}^{V}b_{v}^{m}X_{D}^{v},$$

where $X_{D}={\left[X_{D}^{0},X_{D}^{1},\cdots ,X_{D}^{N_{\mathrm{OFDM}}-1}\right]}^{T}$ represents the input data block, which is partitioned into V disjoint subblocks as $X_{D}={\left[X_{D}^{0},X_{D}^{1},\cdots ,X_{D}^{V-1}\right]}^{T}$. $b_{v}=e^{j\phi_{v}},\;v=1,2,\ldots ,V$ denotes the complex phase factor $b_{v}=e^{j\phi_{v}},\;v=1,2,\ldots ,V$.

The SI blind detection PTS algorithm presented in this paper, which minimizes the accumulation of bit errors, necessitates solely the selection of a candidate weighted phase factor vector, eliminating the requirement for the addition or selection of a corresponding blind detector indicator vector. In this context, the OFDM symbol can be expressed in the form given by Equation (3), where the comb pilot sequence and its positional distribution remain consistent across various OFDM symbols.

For pilot-assisted UWA OFDM systems, define $X_{P}=[X_{P}(0),X_{P}(1),\ldots ,X_{P}(N-1)]$ as the comb pilot sequence. The OFDM symbol can then be represented as follows:

(3) $$X^{m}(k)=X_{D}^{m}(k)+X_{P}(k)=\left\{\begin{array}{c} X_{D}^{m}(k),\;k\in {\mathrm{Po}}^{C}\\ X_{P}(k),\;k\in \mathrm{Po}\;\; \end{array}\right.,$$

where $\mathrm{Po}=\{i_{0},i_{1},\cdots ,i_{N_{p}-1}\}$ represents the position of the pilot subcarrier, and ${\mathrm{Po}}^{C}$ denotes the complement of Po in R=[0,1,⋯ N−1], which corresponds to the position of the data subcarrier.

**Figure 3:**
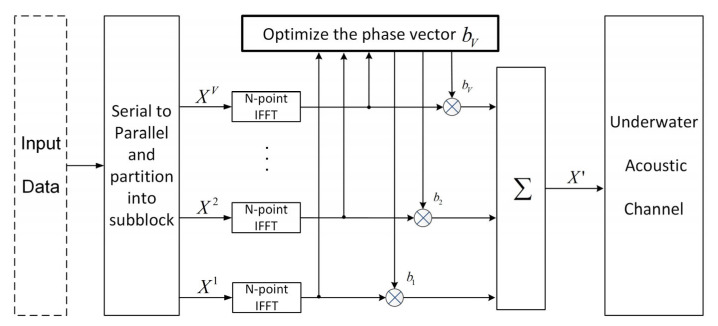
The framework of the PTS technique with SI blind detector based on the minimized error accumulation for PAPR reduction in the UWA coded OFDM system.

The M sets of frequency-domain transmit signals $X^{m}(k)$, after phase rotation, are modulated into the time domain to obtain OFDM candidate symbols $x^{0},x^{1},\cdots ,x^{M-1}$, and the OFDM symbol $x^{\tilde{m}}$ corresponding to PAPR_min is selected as the transmit signal $\tilde{x}$. The principal block diagram of the transmitting end is shown in Figure 3.

The frequency domain data $X_{D}^{m}$ after FFT transformation at the receiver can be written in the following form:

(4) Y(k)=H(k)X(k)+W(k).

The sampled values of the channel frequency domain response can be obtained by

(5) $${\hat{H}}_{P}(k)=\frac{Y_{P}}{X_{P}},$$

where $Y_{P}$ represents the received frequency domain data on the pilot subcarriers. Through channel reconstruction methods such as interpolation and matching pursuit, the complete channel frequency-domain response $\hat{H}(k)$ can be obtained. Depending on system requirements, different equalization criteria are selected. By combining the complete channel frequency domain response $\hat{H}(k)$, the equalized data $X^{\prime }(k)$ are obtained, which represents the data after phase rotation. By performing an inverse phase rotation $X^{\prime }(k)$ with M sets of phase factor vectors $\left\{b_{1},b_{2},\cdots ,b_{v}\right\}$, the de-weighted data ${\tilde{X}}_{e}^{m}$ can be obtained as

(6) $${\tilde{X}}_{e}^{m}=\sum_{v=1}^{V}X_{v}^{\prime }/b_{v}^{m}.$$

**Figure 4:**
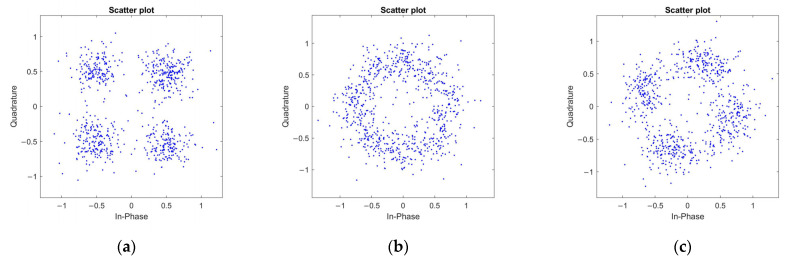
The comparison among the constellations of whether the weighting of the blind detector is correct or not before de-mapping when Eb/N0=16 dB. (**a**) The constellation of the proper weighting; (**b**,**c**) the constellations of the wrong weighting.

Figure 4 presents a comparison of constellation diagrams between the correctly and the incorrectly de-weighted data ${\tilde{X}}_{e}^{m}$. When the equalized data $X^{\prime }(k)$ are de-weighted using the correct weighting phase factor vector, the resulting constellation diagram is shown in Figure 4a. This diagram represents four clusters of constellation points corresponding to QPSK mapping. Figure 4b,c present two typical examples of constellation diagrams after incorrect de-weighting.

In the PTS algorithm based on pseudo-random phase factor vector selection, the candidate phase factor vectors are not limited to the specific set {−1, 1}. Hence, after phase weighting and combining the sub-blocks, the points in the constellation diagram may rotate a certain angle or uniformly rotate into a ring shape. If a hard decision is made for constellation de-mapping of the de-weighted data ${\tilde{X}}_{e}^{m}$ at this stage, nearly half of the data will suffer from de-mapping errors.

The data $X_{D}^{m}(k)$ present on the data sub-carriers are extracted from M groups of ${\tilde{X}}_{e}^{m}$, subsequently undergoing de-mapping to derive ${\tilde{X}}_{c}^{m}$. Then, ${\tilde{X}}_{c}^{m}$ undergoes Viterbi decoding, followed by secondary encoding utilizing the identical convolutional code generation matrix, to obtain ${\tilde{X}}_{rec}^{m}(k)$.

In this scenario, the discrepancy between ${\tilde{X}}_{rec}^{m}(k)$, which is obtained by selecting an incorrect weighted phase factor vector for inverse weighting, and the pre-decoding data ${\tilde{X}}_{c}^{m}$ will be markedly greater than the discrepancy observed when the correct weighted phase factor vector for inverse weighting is chosen. During the process of Viterbi decoding, selecting an incorrect phase factor vector for inverse phase rotation introduces errors that cause data inaccuracies significantly beyond the convolutional code’s capacity for error correction. Consequently, this gives rise to numerous misjudgments and the progressive accumulation of errors throughout the decoding process, thereby perpetuating a vicious cycle in the decoding procedure. The data after secondary encoding will deviate significantly from the original data. Consequently, this phenomenon can be harnessed for achieving blind SI detection, with the decision criterion being formulated in the form of Equation (7).

(7) $$\gamma^{j}=\overset{M-1}{\underset{m=0}{\min}}\left\{\mathrm{biterr}\left({\tilde{X}}_{c}^{m},{\tilde{X}}_{rec}^{m}\right)\right\},$$

where the ‘biterr’ means the calculation result of the number of errors between ${\tilde{X}}_{c}^{m}$ and ${\tilde{X}}_{rec}^{m}(k)$.

**Figure 5:**
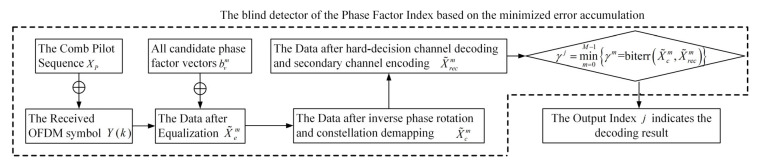
The framework of the SI blind detector based on minimized error accumulation for the UWA coded OFDM system.

The framework of the basic principle of the SI blind detector based on minimized error accumulation is shown in Figure 5. When the storage space in the actual application system is sufficient, all decoding results of the M group can be saved, and the decoding result corresponding to the value j can be selected as the final decoding output. When storage space is limited, the sequence number of the phase factor vector corresponding to j can be chosen, and subsequent processing, such as inverse phase rotation and decoding, can be performed on the equalized received data $X^{\prime }(k)$, thereby obtaining the original transmitted signal.

In coded underwater acoustic OFDM systems, the iterative decoding and re-encoding process using convolutional codes introduces additional computational latency. This latency depends heavily on the performance of the signal-processing platform and the efficiency of the algorithm implementation. On mainstream DSPs such as the Texas Instruments C6000 series—for example, the TMS320C6713 (Texas Instruments, Dallas, TX, USA)—the total time required for one complete Viterbi decoding and re-encoding cycle of a convolutional code with moderate complexity (e.g., constraint length seven and code rate 1/2) is on the scale of tens of microseconds. Thus, for a sub-block count of four, performing eight such iterations would result in a total additional computational latency of approximately several milliseconds. This overhead is generally acceptable compared to the delay required to decode a full OFDM frame before side information can be extracted, and it satisfies the symbol-level real-time requirements of the system.

## 4. Analysis of the Simulation and Tank Experiment Results

### 4.1. Simulation Results

The parameters utilized for the simulation of the UWA coded OFDM system in this section are presented in Table 1. The generation matrix of convolutional codes is shown in Equation (8).

(8) $$G=\left[\begin{array}{c} g_{1}\\ g_{2} \end{array}\right]=\left[\begin{array}{ccccccc} 1 & 1 & 1 & 1 & 0 & 0 & 1\\ 1 & 0 & 1 & 1 & 0 & 1 & 1 \end{array}\right].$$

The length of the encoded code should align with the actual information capacity that a single OFDM symbol can accommodate. To accurately depict the PAPR value of the OFDM symbol and prevent the omission of peak values, an oversampling factor of α=4 is consistently applied to the OFDM signal throughout this chapter. In the simulation, the C-PTS algorithm is designated as the control group. To guarantee equivalency between the error rate of sideband information in the C-PTS algorithm and the bit error rate of other system symbols, the C-PTS algorithm employs identical encoding and mapping techniques for both SI information and data information bits. Additionally, the OFDM symbols conveying SI information are appended to the data OFDM symbols for transmission.

**Figure 6:**
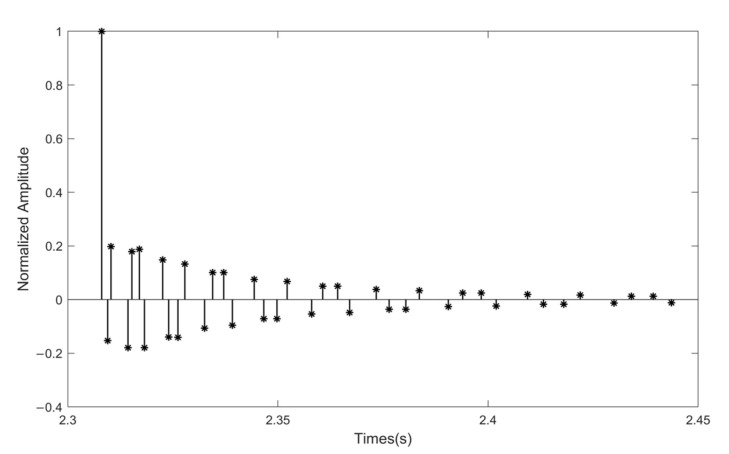
The channel impulse response of the UWA simulation channel.

A dedicated channel generation software was utilized to produce the underwater acoustic multipath channel for the simulation. The sound velocity gradient distribution utilized is representative of typical surface sound channels. Both the transducer and the hydrophone are situated 8 m beneath the sea surface, maintaining a separation distance of 3 km. The channel impulse response (CIR) is shown in Figure 6.

**Figure 7:**
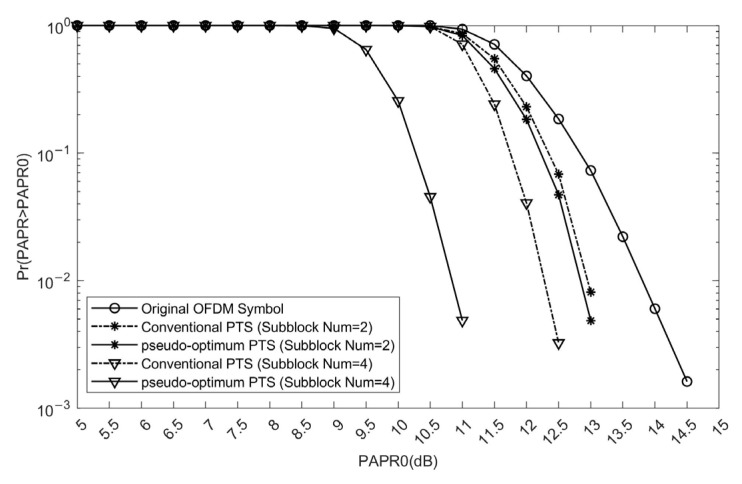
The PAPR reduction performance comparison of the original OFDM signal, the C-PTS scheme, and the pseudo-optimum PTS scheme.

The proposed scheme utilizes the pseudo-optimum PTS method introduced in Reference [23], which offers improved PAPR reduction performance with reduced computational complexity. When the number of sub-blocks is 2, the difference in PAPR suppression performance between the pseudo-random and conventional algorithms is marginal; however, as the number increases, their performance diverges significantly. Under identical configurations of sub-blocks (Subblock Number = 4) as the C-PTS scheme, the pseudo-optimum PTS achieves a gain of approximately 3.5 dB compared to the original OFDM signal, and outperforms the C-PTS scheme by about 1.5 dB, as illustrated in Figure 7.

In the subsequent part of this section, we only compare the SI accuracy and the system BER performance of the proposed scheme against the C-PTS algorithm.

In coded OFDM systems, the bit error rate (BER) performance is notably influenced by the signal-to-noise ratio (SNR). Consequently, when the SNR is low, the $\gamma^{j}$ will be relatively indistinguishable, irrespective of whether the correct phase factor vector is used for inverse phase rotation of the received data, rendering it challenging for blind detectors to distinguish, and thus hard to provide the perfect SI.

**Figure 8:**
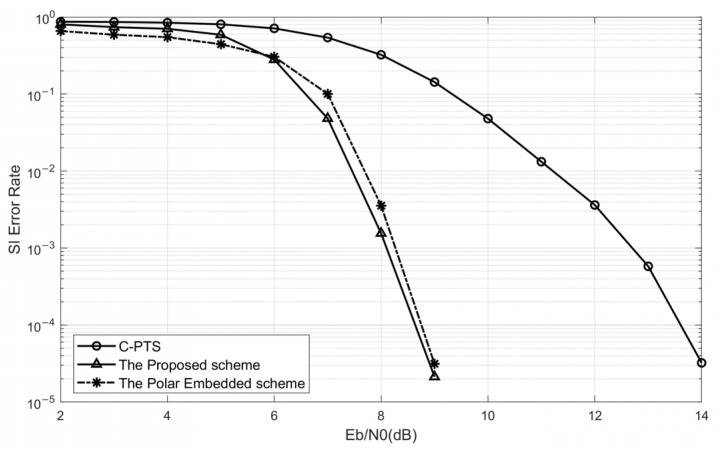
The comparisons of the SI error rate between the C-PTS, the embedded SI transmission scheme based on polar code, and the PTS with an SI blind detector based on the minimized error accumulation.

Figure 8 compares the curves of the SI error rate versus $Eb/N_{0}$ for the C-PTS scheme and the PTS scheme based on minimized error accumulation. When $Eb/N_{0}$ is less than 6 dB, the curve exhibits a slower rate of decline, attributable to the constraint that the SNR significantly impacts the BER performance. Nevertheless, as illustrated in Figure 8, when $Eb/N_{0}$ exceeds 6 dB, the convergence speed of the SI error rate for the proposed scheme in this paper surpasses that of the C-PTS algorithm by a considerable margin. When $Eb/N_{0}$ exceeds 9 dB, the SI can be precisely identified without any errors, whereas the C-PTS algorithm requires a value of 15 dB to ensure error-free transmission of the SI.

Figure 8 also compares the SI error rate between the embedded SI transmission scheme based on polar code in Reference [26] and the proposed algorithm. The results demonstrate that the performance of the proposed algorithm is comparable to that of the SI-embedded polar code algorithm. Both algorithms achieve error-free SI reception when $Eb/N_{0}$ exceeds 9 dB, thus meeting the requirements for reliable underwater acoustic communication.

**Figure 9:**
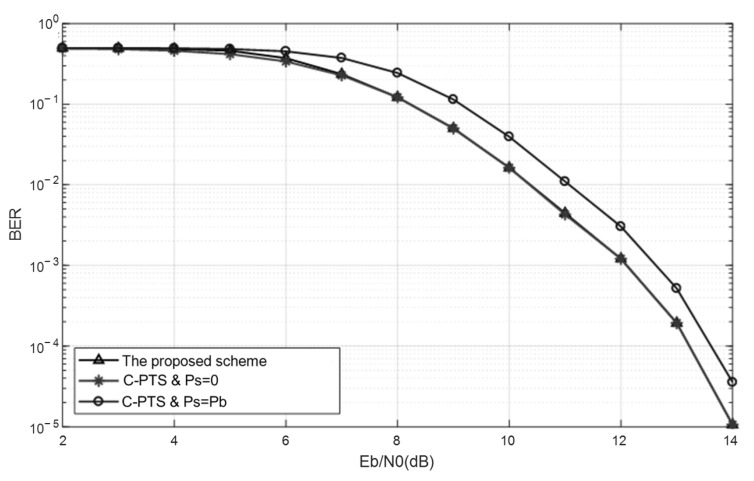
The overall BER performance comparison between the C-PTS and the PTS with an SI blind detector based on minimized error accumulation.

Figure 9 presents a comparative analysis of the system BER performance among the proposed PTS scheme, the C-PTS algorithm, and the C-PTS algorithm augmented with prior Side Information. From the BER curve with $P_{s}=0$ in Figure 9, it becomes evident that when $Eb/N_{0}<9\mathrm{dB}$, even with $P_{s}=0$, the BER persists within the range of $10^{-1}$ to $10^{-2}$. Under such circumstances, the UWA OFDM system struggles to meet the requirements for reliable communication. Hence, the existence of the SI error rate has no bearing on the actual system performance. Despite a slight discrepancy between the BER curves of the SI-known PTS algorithm and the proposed scheme when $Eb/N_{0}<9\mathrm{dB}$, the difference is insignificant, rendering the impact of SI error on the system BER performance negligible. Notably, the curves of the two algorithms virtually converge completely when $Eb/N_{0}>9\mathrm{dB}$. By examining the SI error rate curve of the proposed scheme in Figure 8, it can be deduced that the proposed scheme is capable of furnishing precise side information to the receiver when the SNR attains a sufficient level for ensuring reliable communication. However, owing to the persistent SI error rate, the BER curve of the C-PTS algorithm consistently exhibits a notable disparity when compared to the BER curve with $P_{s}=0$. The analysis mentioned above demonstrates that the SI blind detector in the proposed scheme surpasses the C-PTS algorithm in terms of the system BER performance, ensuring reliable communication.

The algorithm proposed in this paper also has several limitations. For instance, the SI error rate increases under low SNR conditions. However, since reliable underwater acoustic communication generally requires moderate to high SNR, operation at extremely low SNRs (e.g., $Eb/N_{0}$ below 8 dB) is challenging regardless of the SI transmission or detection scheme. Thus, the degraded performance of the proposed algorithm in such conditions is considered acceptable.

Additionally, the multiple decoding and re-encoding steps with convolutional codes introduce additional computational complexity. Nonetheless, since DSPs often feature built-in convolutional codecs, the resulting overhead is generally manageable.

In the case of high-order modulation, the minimum $Eb/N_{0}$ required to achieve near-zero SI error increases with the modulation order. Nevertheless, since high-order modulation itself exhibits poorer error performance compared to lower-order schemes, the proposed algorithm remains capable of providing reliable side information at SNR levels where such modulation systems can operate effectively.

### 4.2. Field Experiment Results

A field experiment was conducted to verify the reliability of the proposed scheme at the Huanghai Sea, Qingdao, China. During this experiment, the boats holding the transducer and the hydrophone were both anchored. The transducer and the hydrophone were submerged at depths of 7 m and 4 m, respectively. During data transmission, the distance between the transmitting boat and the receiving boat was approximately 1.05 km, with sea state 3. During this experiment period, the receiving boat was located in proximity to the primary shipping route, where numerous fishing vessels frequently passed by, leading to high noise energy in the received signal. The received data exhibited a signal-to-noise ratio (SNR) of approximately 9.7 dB. During the experiments, the built-in audio interface of a laptop was used as the signal source to generate OFDM signals. A power amplifier amplified these signals and then transmitted them through an acoustic projector with an operating frequency band of 8–16 kHz. At the receiver side, an 8105-type hydrophone was used for signal reception and data acquisition.

**Figure 10:**
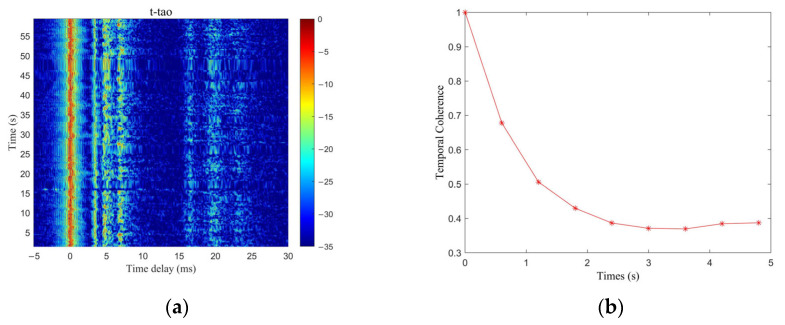
The real impulse response of the time-varying channel of the Yellow Sea. (**a**) The channel impulse response of the field channel as a function of time; (**b**) the temporal coherence of the time-varying channel of the Yellow Sea.

The time-dependent CIRs of the field channel are given in Figure 10a. The estimation of the channel’s temporal coherence can be derived by utilizing the CIR measurements at various time points as the reference signal, as illustrated in Figure 10b. Due to the unsettled sea surface, the temporal coherence diminishes rapidly, rendering each symbol’s experienced channel effectively independent.

**Figure 11:**
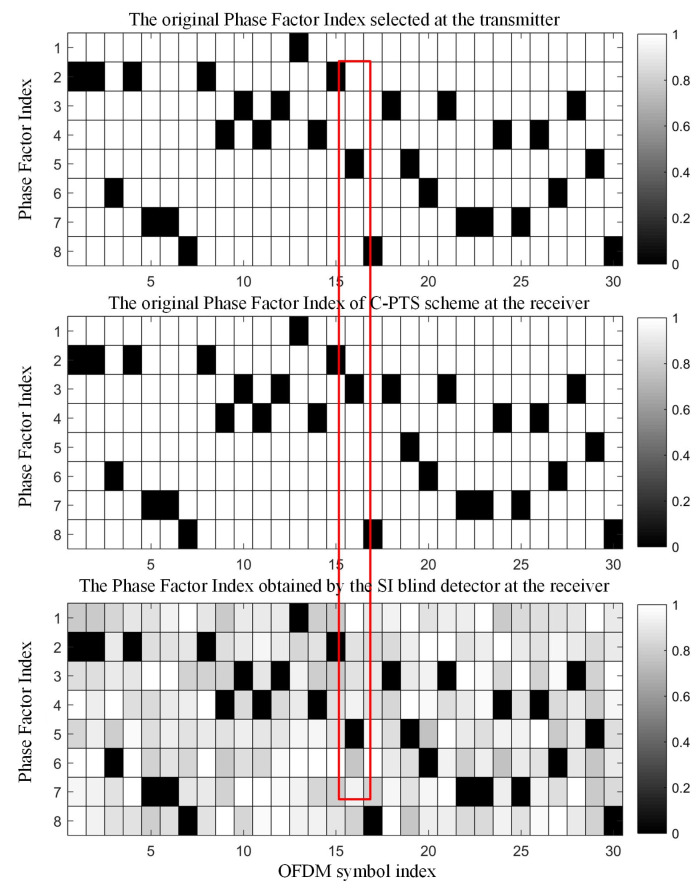
Comparison of the SI detection result between the C-PTS and the SI blind detector.

Take the first 30 OFDM symbols from this experiment as an example. Figure 11 presents a comparison between the decoding results of the SI transmission in the C-PTS scheme and the autonomous recognition results of the SI blind detector. A gray value of 0 in the top two diagrams indicates the index of the selected phase factor vector. The bottom diagram displays the output results of autonomous recognition by the SI blind detector, which is based on minimized error accumulation. The gray value in the diagram is the decision value mentioned in Equation (8), and the minimum gray value corresponds to the decision output of the SI blind detector.

The C-PTS algorithm encounters decoding errors in the SI at the OFDM symbols highlighted in red boxes, owing to the influence of multipath channels and ship noise. Conversely, the output of the SI blind detector positioned at the bottom remains in accordance with the SI information transmitted by the transmitter. It is demonstrated that the SI blind detector proposed in this paper can accurately distinguish the index of the phase factor vector when the received signal-to-noise ratio is 10 dB, ensuring the reliability of the underwater acoustic OFDM communication system.

**Figure 12:**
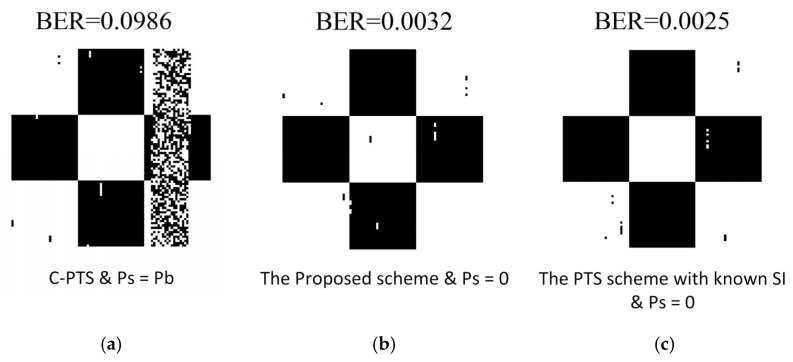
Transmitted figure results comparison of the field experiment. (**a**) The transmitted figure result of the C-PTS scheme; (**b**) the transmitted figure result of the proposed scheme; (**c**) the transmitted figure result of the scheme with known SI.

Figure 12 contrasts the outcomes of sea trial image transmission employing the C-PTS algorithm (Figure 12a), the proposed PTS scheme (Figure 12b), and the PTS scheme with known SI (Figure 12c). Typically, the transmitted data will be fully interleaved to avoid continuous errors. In this experiment, a black-and-white checkerboard pattern is used as the transmission data, and it is not interleaved. The purpose is to fully demonstrate the scenario of full-symbol errors in OFDM symbols caused by the SI errors. When a striped error pattern appears in the figure, it means that an SI transmission error has occurred.

Notably, there exists a striping error in Figure 12a, which the mistranslated side information causes. Due to mistakes in SI, an entire OFDM symbol was decoded incorrectly, resulting in the occurrence of stripe errors in the image. The BER performance of the UWA OFDM systems will possibly be ruined since any error in the detection of side information can damage the entire data symbols. Thus, the mistakes in SI contribute to overall symbol errors in the C-PTS algorithm, which requires the SI transmission, yielding a BER discrepancy of roughly two orders of magnitude in comparison to the enhanced PTS algorithm featuring the SI autonomous identification. The BER performance of the proposed algorithm is comparable to that of the known SI algorithm, with the BER being in the same order of magnitude, which shows that the proposed algorithm can provide accurate side information for the receiver.

This maritime trial experiment verifies that the proposed PTS scheme, which employs an SI blind detector based on minimized error accumulation, is capable of achieving reliable communication in the complex multipath channel UWA communication scenarios.

## 5. Conclusions

This paper proposed a blind SI detector based on minimized error accumulation, suitable for the coded UWA OFDM systems. This algorithm utilizes the error correction capability tolerance during the channel encoding and decoding process. After conducting the inverse phase rotation by the wrong phase vector, the constellation exhibits a rotation, which will lead to error accumulation during the channel decoding process. The SI autonomous identification can be achieved by comparing the BER between the post-demapping data and the decoded and recoded data. The proposed scheme in this paper provides accurate SI information for the receiver under appropriate SNR conditions, avoiding the BER disaster caused by the SI errors in the C-PTS scheme. The simulation and field experiment results also demonstrated that the BER performance of the proposed scheme is approximately identical to that of PTS with perfect SI, indicating that the SI provided by the blind detector is excellent. It is suitable for coded UWA OFDM communication systems that use hard decision results as the input for decoding. In summary, the SI blind detection PTS algorithm proposed in this paper changes the transmission and judgment methods of side information in traditional algorithms, breaks away from the dependence of BER performance on side information transmission in conventional methods, improves the frequency band utilization rate of the UAC OFDM systems, and ensures the real-time performance and reliability of the system.

## Figures and Tables

**Table 1:** Simulation parameters of the UWA OFDM system.

Parameters	Value
Transmission frequency band	8–16 kHz
Sampling frequency	48 kHz
Number of total subcarriers	1024
Subcarrier bandwidth	7.8125 Hz
Oversampling rate	4
Constellation mapping scheme	QPSK modulation
Pilot spacing	4
Subblock number	4
Number of phase factor vectors	M=8
Code rate of the convolutional code	1/2

## Data Availability

The data analyzed during the current study are available upon reasonable request from the corresponding author.

## References

[1] Yang J., Wang J., Qiao G., Liu S., Ma L., He P. (2024). Review of Underwater Acoustic Communication and Network Technology. J. Electron. Inf. Technol..

[2] Adil M., Liu S., Mazhar S., Alharbi A., Yan H., Muzzammil M. (2025). A Novel Transfer Learning-Based OFDM Receiver Design for Enhanced Underwater Acoustic Communication. J. Mar. Sci. Eng..

[3] Qu F., Fu Y., Yang S., Zhuo X., Tu X., Wei Y. (2023). An overview of the development status of underwater acoustic communication technology applied to ocean Internet-of-things. J. Harbin Eng. Univ..

[4] Xiao S., Liu T., Liu B., Zhang Y. (2025). Adaptive algorithm for compressed sensing channel estimation in underwater acoustic OFDM systems. Phys. Commun..

[5] Ma L., Li M., Liu S., Ma Z., Wu H. (2022). A multi-beam space diversity method for long-range underwater acoustic OFDM communication in deep water. Acta Acust..

[6] Zhao H., Wang B., Zhang L., Zheng L. (2025). MMFFNet: Multi-Node Fusion Network for Passive Fusion Detection of Underwater Acoustic Communication Signals. IEEE Commun. Lett..

[7] Kim K.H. (2022). Dither Signal Design for PAPR Reduction in OFDM-IM over a Rayleigh Fading Channel. IEICE Trans. Commun..

[8] Murad M., Tasadduq I.A., Otero P. (2022). Ciphered BCH Codes for PAPR Reduction in the OFDM in Underwater Acoustic Channels. J. Mar. Sci. Eng..

[9] Wu J., Ma X., Yin Y., Babar Z. (2017). A novel algorithm to mitigate the effect of clipping in orthogonal frequency division multiplexing underwater communication acoustic sensor system. Int. J. Distrib. Sens. Netw..

[10] Yang L., Song K., Siu Y. (2017). Iterative Clipping Noise Recovery of OFDM Signals Based on Compressed Sensing. IEEE Trans. Broadcast..

[11] Liu G., Liu X. (2011). Research on the Reduction of PAR in Underwater OFDM Acoustic Communication by Combined Method. Mod. Electron. Tech..

[12] Banoori F., Shi J., Khan K., Arshad J., Liu X., Noman S.M., Irshad M. (2023). Energy efficiency augmentation in UWA-OFDM transducer by peak to average power ratio alleviation through hybrid companding approach. Trans. Emerg. Telecommun. Technol..

[13] Lee B.M., Rim Y.S., Noh W. (2017). A combination of selected mapping and clipping to increase energy efficiency of OFDM systems. PLoS ONE.

[14] Wu J. (2018). Iterative Compressive Sensing for the Cancellation of Clipping Noise in Underwater Acoustic OFDM System. Wirel. Pers. Commun..

[15] Bauml R.W., Fischer R. (1996). Reducing the peak-to-average power ratio of multicarrier modulation by selected mapping. Electron. Lett..

[16] Shi N., Liu X., Zhou L., Zhang H., Xiong J., Zhao H., Wei J. (2024). Peak-to-Average Power Ratio Reduction Using Selected Mapping for Mixed Numerology NOMA. IEEE Trans. Wirel. Commun..

[17] Gunturu C., Valluri S.P. (2025). Complexity reduction strategies in selected mapping-based direct current-biased optical orthogonal frequency division multiplexing systems. Opt. Eng..

[18] Muller S.H., Huber J.B. (1997). OFDM with reduced peak-to-average power ratio by optimum combination of partial transmit sequences. Electron. Lett..

[19] Jawhar Y.A., Audah L., Taher M.A., Ramli K.N., Shah N.A.M., Musa M., Ahmed M.S. (2019). A Review of Partial Transmit Sequence for PAPR Reduction in the OFDM Systems. IEEE Access.

[20] Abraham D., Jose R., Manuel M. (2024). Sparse Ramanujan Sequences Transform based OFDM for PAPR reduction. IETE J. Res..

[21] Kwon O.J., Ha Y.H. (2003). Multi-Carrier PAPR reduction method using Sub-optimal PTS with threshold. IEEE Trans. Broadcast..

[22] Cimini L.J., Sollenberger N.R. (2000). Peak-to-Average Power Ratio Reduction of an OFDM Signal Using Partial Transmit Sequences. IEEE Commun. Lett..

[23] Xing S., Qiao G., Ma L. (2018). A Blind Side Information Detection method for Partial Transmitted Sequence Peak-to-Average Power Reduction Scheme in OFDM Underwater Acoustic Communication System. IEEE Access.

[24] Hussain I.M., Tasadduq I.A. PAPR reduction of OFDM signals using autocorrelation based SLM without side information. Proceedings of the 2008 Canadian Conference on Electrical and Computer Engineering.

[25] Joo H.S., Kim K.H., No J.S., Shin D.J. (2017). New PTS Schemes for PAPR Reduction of OFDM Signals without Side Information. IEEE Trans. Broadcast..

[26] Xing S., Wei B., Yu Y., Gong X. (2024). A Novel Embedded Side Information Transmission Scheme Based on Polar Code for Peak-to-Average Power Ratio Reduction in Underwater Acoustic OFDM Communication System. Sensors.

[27] Du Z., Beaulieu N.C., Zhu J. (2009). Selective Time-Domain Filtering for Reduced-Complexity PAPR Reduction in OFDM. IEEE Trans. Veh. Technol..

[28] Guan L., Jiang T., Qu D., Zhou Y. (2010). Joint Channel Estimation and PTS to Reduce Peak-to-Average-Power ratio in OFDM System without Side Information. IEEE Signal Process. Lett..

[29] Wang W., Xing S., Qiao G. (2013). A selective mapping peak-to-average power ratio reduction algorithm without side information for underwater acoustic multiple-input multiple-output orthogonal frequency division multiplexing communication. Acta Phys. Sin..

[30] Qiao G., Xing S., Zhou F. (2021). Selected-Mapping Peak-to-Average Power Reduction Method with Orthogonal Pilot Sequences in Underwater Acoustic OFDM System Without Side Information. Chin. J. Electron..

